# Alternative Splicing of the Pituitary Adenylate Cyclase-Activating Polypeptide Receptor PAC1: Mechanisms of Fine Tuning of Brain Activity

**DOI:** 10.3389/fendo.2013.00055

**Published:** 2013-05-21

**Authors:** Janna Blechman, Gil Levkowitz

**Affiliations:** ^1^Department of Molecular Cell Biology, Weizmann Institute of ScienceRehovot, Israel

**Keywords:** ADCYAP1R1, activity-dependent gene regulation, zebrafish model system, PACAP receptor, stress disorders, post-traumatic, hypothalamic hormones, homeostasis

## Abstract

Alternative splicing of the precursor mRNA encoding for the neuropeptide receptor PAC1/ADCYAP1R1 generates multiple protein products that exhibit pleiotropic activities. Recent studies in mammals and zebrafish have implicated some of these splice isoforms in control of both cellular and body homeostasis. Here, we review the regulation of PAC1 splice variants and their underlying signal transduction and physiological processes in the nervous system.

## Introduction

G-protein-coupled receptors (GPCRs) represent the largest family of membrane-associated proteins mediating physiological responses in vertebrates by means of controlling metabolic, neural, and developmental functions (Lefkowitz, 2007; Frooninckx et al., 2012; Zhang and Eggert, 2013). These proteins are expressed in almost all types of tissues and are represented by over 1,000 membrane receptors for extracellular (EC) ligands including hormones, neurotransmitters, pheromones, lipids, and other proteins (Xue et al., 2008; Markovic and Grammatopoulos, 2009; Nordstrom et al., 2009; Hoyer and Bartfai, 2012; Jafurulla and Chattopadhyay, 2013). The basic structure of GPCR proteins consists of a seven-transmembrane (TM) domain with a complex EC structure composed of an N-terminal region and three EC loops involved in the diverse ligand recognition process. Three intracellular (IC) loops and a C-terminal domain transduce a signal into the cell’s cytoplasm and nucleus. Typically, ligand binding to the EC loops induces conformational changes in the TM and IC domains of the receptor resulting in specific coupling to a set of cytoplasmic molecules, termed G-proteins, each composed of different isoforms of alpha, beta, and gamma subunits. G-proteins in turn regulate the activity of IC effector molecules, including adenylate cyclase (AC), phospholipase Cβ (PLC), and RhoGEF causing the activation of secondary messengers such as cyclic AMP (cAMP), inositol-1,4,5-triphosphate (InP3), and diacylglycerol leading to the initiation of distinct IC signaling pathways (Simon et al., 1991; Sah et al., 2000; Pierce et al., 2002; Oldham and Hamm, 2008; Mizuno and Itoh, 2009; Mahata et al., 2011; Maurice et al., 2011).

The diversity of the GPCR-initiated signal transduction pathways is determined by the multiplicity of cognate ligands with different receptor binding properties and by the receptors’ membrane-interacting partners that form homo- and hetero-dimerized GPCR complexes. These receptor dimers have unique binding properties to both EC ligands and a set of IC G-proteins (Milligan and Kostenis, 2006; Harikumar et al., 2008; Furness et al., 2012). Thus, ligand binding, along with receptor heterodimerization, can generate diversity in G-proteins coupling that can generate varied IC signaling functions.

Beyond the complexity of the aforementioned EC and IC GPCRs interacting effectors, the genetic diversity of the GPCR family can be generated by means of alternative precursor mRNAs (pre-mRNA) splicing of exons encoding specific protein moieties and causing considerable functional differences of the resulting splicing products (Black, 2003; Jaillon et al., 2008; Furness et al., 2012). Alternative splicing mechanisms allow the generation of multiple mRNA transcript variants from a single gene by utilizing different combinations of exons by means of skipping or insertion of alternatively spliced exons. In particular, neuronal cells are known to exhibit high levels of alternative splicing, generating the basis for molecular and cellular diversity important for the patterning and maintenance of the central and peripheral nervous systems (Yeo et al., 2007; Betke et al., 2012; Norris and Calarco, 2012; Sun et al., 2012). In the case of GPCRs, this combinatorial exon assembly can lead to changes in the protein domains responsible for ligand binding, IC effector coupling, as well as receptor stability, and endocytosis.

In this review we focus on one GPCR, the pituitary AC-activating polypeptide (PACAP) receptor (ADCAYP1R1), also known as PAC1. This receptor represents a fascinating example of how alternative splicing of a single GPCR gene leads to different physiological outcomes. We will describe the current knowledge regarding the role and mechanism of action of PAC1 splice variants in development, physiology, and diseases focusing on PAC1’s role in the nervous system.

## The PAC1 Receptor

PAC1 belongs to the glucagon/secretin receptor family of GPCRs that consists of hormone and neuropeptide receptors. These receptors signal through coupling to the G-protein alpha subunits Gs and Gq that typically activate AC and phospholipase Cβ enzymes, respectively (McCulloch et al., 2002; Ahren, 2008; Dickson and Finlayson, 2009; Vallejo, 2009; Vaudry et al., 2009). Coupling of PAC1 to Gs may also lead to cAMP-dependent accumulation of IC calcium (Braas and May, 1996; Mustafa et al., 2010). The PAC1 gene contains multiple exons that undergo extensive alternative splicing. The peptide PACAP is the high-affinity ligand for PAC1. Post-translational proteolytic processing of the PACAP precursor protein generates several polypeptides with varying sizes, including PACAP38 and PACAP27 (Miyata et al., 1989; Vaudry et al., 2009; Harmar et al., 2012; Watkins et al., 2012). PAC1 was found to play a pivotal role in the spatio-temporal regulation of proliferation, differentiation, or cell survival during development as well as in the regulation of synthesis and release of neuroendocrine hormones.

**Figure 1:**
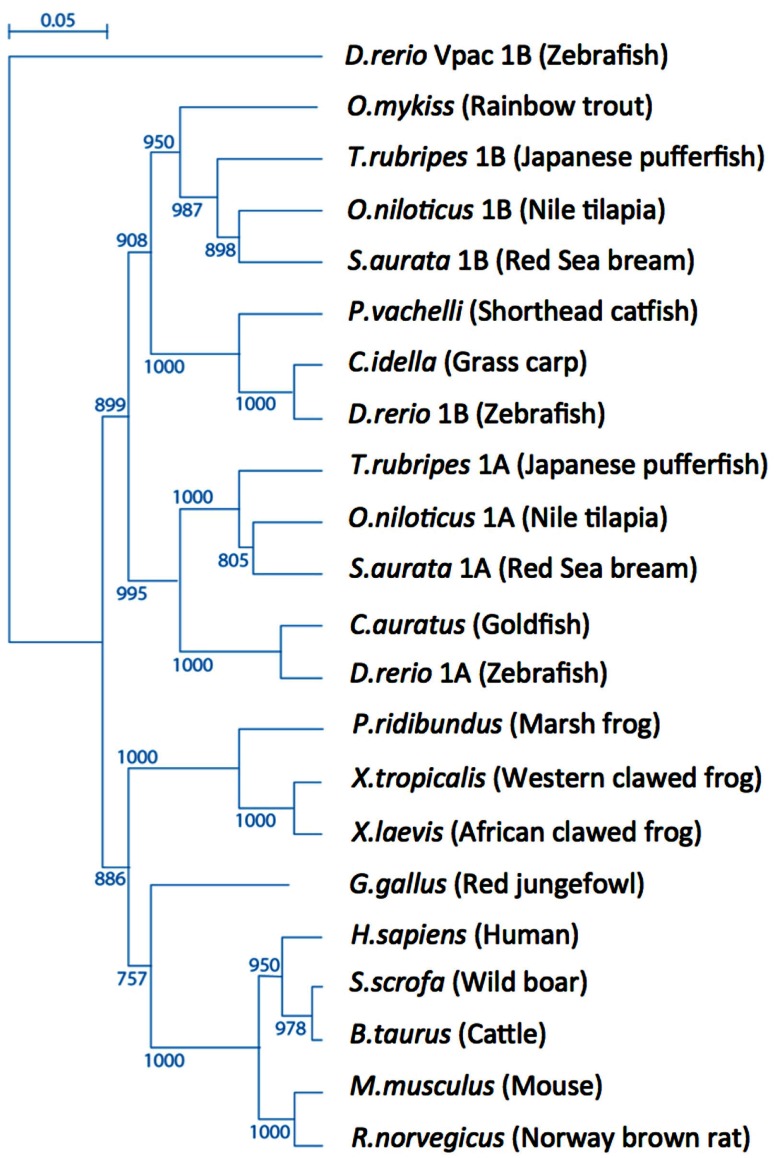
**Tree topology of the vertebrate PAC1 receptor family**. Phylogenetic relationships of PAC1 receptors analyzed using the zebrafish (*Danio rerio*) Vpac1b gene as an out-group.

Phylogenetic analysis of the vertebrate PAC1 receptor family indicates their origin from a common ancestral gene and demonstrates a tree topology with species-specific paralogs belonging to different separated sub-groups (Figure 1). Bony fishes (*teleosts*) genomes typically contain more than one gene due to teleost-specific gene duplication event of both ligand and receptor molecules during the evolution of these species (Wei et al., 1998; Fradinger et al., 2005; Bjarnadottir et al., 2006; Cardoso et al., 2007; Machluf et al., 2011).

## Alternatively Spliced PAC1 Gene Products

**Figure 2:**
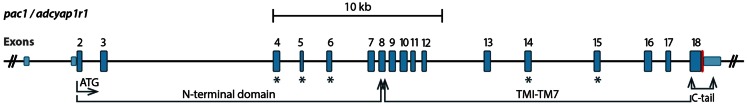
**A diagram showing genomic organization of the *PAC1* (*ADCYAP1R1*) gene**. Exons 2–18 encode the open reading frame. Exons undergoing the alternative splicing discussed in this review are marked with blue asterisks. Exon 4, 5, and 6 encode parts of the N-terminal domain, whereas exons 14 and 15 encode the third intracellular loop. TM, transmembrane.

**Figure 3:**
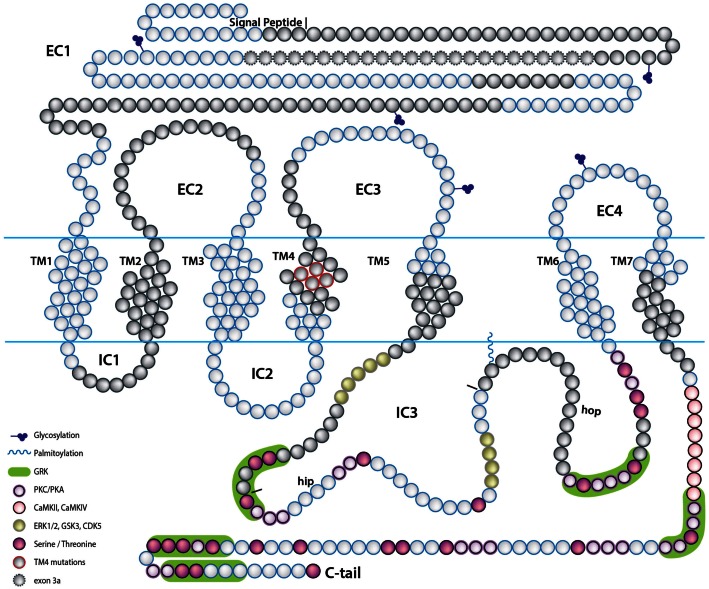
**A scheme depicting the topology of the PAC1 receptor**. The relative location of amino acids encoded by PAC1 exons are depicted by the blue and gray beads. Predicted PAC1 structural motifs known to be involved in protein-protein interactions determining G-protein mediated PAC1 signaling are marked on the protein diagram with regard to putative motifs formed due to insertions of hop and hip cassettes into the IC3 loop sequence. EC, extracellular loop; IC, intracellular loop; TM, transmembrane domain.

The alternative splicing of PAC1 has been extensively studied. In this review we use the exon numbers designation of the human *PAC1* and otherwise indicate cases where the exons numbers do not match. The human gene was shown to contain 18 exons with the open reading frame encoded by exons 2–18 (Chatterjee et al., 1997; Lutz et al., 2006). Ten of these exons are constitutively expressed (exons 2, 3, 7–13, 18), whereas the rest (exons 4–6, 14, 15, and possibly 16, 17) are regulated by the alternative splicing (Figure 2). The N-terminal part of PAC1’s EC domain is encoded by six exons. Exons 7–17 encode the seven-TM domains including EC and IC loops and exon 18 encodes the C-terminal cytoplasmic tail including the 3′-untranslated region (Figures 2 and 3). PAC1 splicing variants were identified in other vertebrate species including rat, mouse, frog, and fish. In Table 1 we assembled all known PAC1 splice isoforms from different vertebrate species. The functional outcome of these splicing events will be discussed later. By and large, PAC1 alternative splicing can be divided into four types of splicing events that impact receptor functions (Figure 3; Table 1): (1) Variations in the EC N-terminal domain altering the ligand-binding specificity and affinity. (2) Variations in exons encoding to part of the third IC loop (IC3) thereby affecting G-protein coupling and/or interaction with other IC signaling proteins. (3) Variations in the TM domains TM2 and TM4 contributing to the receptors heteromerization and IC transport. (4) Variations in the 5′ UTR that may affect mRNA expression dynamics. Notably, splicing products containing different combinations of N-terminal and IC3 splice variants were identified in mammalian species.

**Table 1:** **Biochemical properties of PAC1 splice variants**.

PAC1 isoforms	Specie	Sequence alterations	Binding properties	Signaling consequences			Reference
				AC activation (Gs) cAMP production	PLC stimulation (Gq) IP3 turnover	Ca^2+^ mobilization from intracellular and extracellular stores	
null	rat, human mouse* zebrafish	PAC1 containing exons 2–13, 16–18	Pacap38 ≈ Pacap27 ≫ VIP Pacap38 ≫ Pacap27 ≫ VIP*	Pacap38 ≤ Pacap27 ≫ VIP Pacap38 ≤ Pacap27 ≫ VIP*	Pacap38 ≫ Pacap27 > VIP	Pacap38 > Pacap27 ≫ VIP Pacap38 > Pacap27 ≫ VIP* Pacap ≈ VIP (ES cells)	Spengler et al. (1993), Wei et al. (1998), Grumolato et al. (2003), Fradinger et al. (2005), Lutz et al. (2006), Ushiyama et al. (2007, 2010), Chafai et al. (2011), Holighaus et al. (2011)
hop1 SV2*	rat, mouse human*	Insertion of exon 15 into IC3 loop	Pacap38 ≈ Pacap27 ≫ VIP	Pacap38 ≤ Pacap27 ≫ VIP Pacap38 ≤ Pacap27*	Pacap38 ≫ Pacap27 ≈ VIP Pacap38 ≥ Pacap27* Pacap38 ≈ Pacap27 ≫ VIP (SCG)	Pacap38 > Pacap27 ≫ VIP ND*	Spengler et al. (1993), Pisegna and Wank, 1996, Pisegna et al. (1996), McCulloch et al. (2002), Ronaldson et al. (2002), Mustafa et al. (2007), May et al. (2010), Holighaus et al. (2011)
hop1 novel	rat	193 bp deletion: TM6, EC3 loop, TM7, and part of C-terminal tail	Pacap38 ≈ Pacap2 ≫ VIP	No activation – Arg416 and Ser417 are essential for G-protein coupling	No activation	ND	Abu-Hamdan et al. (2006)
hop2	rat zebrafish*	Insertion of exon 15 into IC3 loop	Pacap38 ≈ Pacap27 ≈ VIP Pacap38 > Pacap27 ≫ VIP*	Pacap38 ≤ Pacap27 Pacap38 ≈ VIP Pacap38 < Pacap27 ≫ VIP*	Pacap38 ≫ Pacap27 ND*	Pacap38 ≫ VIP ND*	Spengler et al. (1993), Cavallaro et al. (1995), Wei et al. (1998), Grimaldi and Cavallaro (1999), Pilzer and Gozes (2006b)
hip SV1*	rat human*	Insertion of exon 14 into IC3 loop	Low affinity Pacap38 ≥ VIP Pacap38 ≈ Pacap27 ≫ VIP*	Low potency Pacap38 < Pacap27 ≈ VIP Pacap38 ≤ Pacap27*	No activation Pacap38 > Pacap27 ≈ VIP*	No Ca^2+^ influx ND*	Spengler et al. (1993), Journot et al. (1995), Pisegna and Wank, 1996, Pisegna et al. (1996), Ronaldson et al. (2002), Germano et al. (2004), Lutz et al. (2006)
hip-hop SV3*	rat, human*	Insertion of exons 14, 15 into IC3 loop	Pacap38 ≈ Pacap27 ≫ VIP	Low potency Pacap38 < Pacap27 ≈ VIP Pacap38 ≤ Pacap27*	Pacap38 ≫ Pacap27 Pacap38 ≥ Pacap27*	No Ca^2+^ influx ND*	Spengler et al. (1993), Pisegna and Wank, 1996, Pisegna et al. (1996), Lu et al. (1998), Abu-Hamdan et al. (2006)
short δ5,6*	rat, mouse, human*	Deletions of exons 5,6	Pacap38 ≈ Pacap27 ≥ VIP Pacap38 ≈ Pacap27 > VIP*	Pacap38 ≈ Pacap27 ≥ VIP Pacap38 ≈ Pacap27 > VIP*	Pacap38 ≈ Pacap27 > VIP	Pacap38 > Pacap27 ≫ VIP	Pantaloni et al. (1996), Dautzenberg et al. (1999), Lutz et al. (2006), Ushiyama et al. (2007, 2010)
short hop1 δ5,6 hop1*	rat, mouse, human*	Deletions of exons 5,6, insertion of exon 15	Pacap38 ≈ Pacap27 ≫ VIP ND*	Pacap38 > Pacap27 ≫ VIP Pacap38 ≥ VIP*	Pacap38 > Pacap27 ≫ VIP Pacap38 > VIP*	ND Pacap38 > Pacap27 ≫ VIP*	Dautzenberg et al. (1999), Lutz et al. (2006), Ushiyama et al. (2007, 2010)
δ5,6-hip	human	Deletions of exons 5,6, insertion of exon 14	Pacap38 ≈ VIP	Low potency Pacap38 ≈ VIP	Low potency Pacap38 ≥ VIP	ND	Lutz et al. (2006)
very short δ4,5,6*	rat, human*	Deletions of exons 4,5,6	Low affinity Pacap38 ≈ Pacap27 > VIP	Low potency Pacap38 ≈ Pacap27 > VIP Pacap38 ≈ Pacap27 ≫ VIP*	No activation	ND Pacap38 > Pacap27 ≫ VIP*	Pantaloni et al. (1996), Dautzenberg et al. (1999), Lutz et al. (2006)
3a	rat	N-terminal insertion of exon 3a (72 bp)	Sixfold increased affinity to Pacap38	Reduced sensitivity to Pacap38; Pacap27 like null	Reduced sensitivity to Pacap38 and Pacap27	Pacap38 and Pacap27 stimulate Ca^2+^ like v null	Daniel et al. (2001), Ajpru et al. (2002), Pilzer and Gozes (2006b)
Pac-TM4	rat	TM4-deletion/insertion (12 to 6 bp), TM2 –D136N in EC1, N190D	Pacap38 ≈ Pacap27	Low potency (BNK cells) Pacap27 ≫ VIP; no activation (CHO cells)	Low potency (BNK) Pacap27 ≫ VIP; no activation (CHO)	Pacap27 via activation of dihydropyridine-sensitive L-type Ca^2+^ channels	Chatterjee et al. (1996), Ajpru et al. (2002)
δ5	human	Deletion of exon 5	Pacap38 ≈ Pacap27 > VIP	Pacap38 ≈ Pacap27 ≫ VIP	Pacap38 ≫ Pacap27 ≈ VIP	ND	Lutz et al. (2006)
δ5hop1	human	Deletion of exon 5, insertion of exon 15	ND	Pacap38 ≈ Pacap27 ≫ VIP	Pacap38 ≫ Pacap27 ≈ VIP	ND	Lutz et al. (2006)
δ5hip	human	Deletion of exon 5, insertion of exon 14	Pacap38 ≥ VIP	Low potency Pacap38 > VIP	No activation	ND	Lutz et al. (2006)
δ5,6,14–17	human	Deletion of exons 5,6, deletion/insertion in TM6, EC3 & TM7	Pacap38 ≈ Pacap27 > VIP	No activation	No activation	ND	Lutz et al. (2006)
skip	zebrafish	Insertion into the IC3 loop with premature stop codon	ND	ND	No activation, loss of G-protein coupling epitope	ND	Fradinger et al. (2005)
R25	frog	Insertion of a hop-like cassette into the IC3 loop	Pacap38 > VIP	Pacap38 > VIP (Pac25 > Pac41 > Pac1-null)	ND	ND	Alexandre et al. (2002)
R41	frog	Insertion into the IC3 loop	Pacap38 > VIP	Pacap38 > VIP	ND	ND	Alexandre et al. (2002)
Rmc	frog	Insertion into TM7 C-term.	Pacap38 > VIP	Prevents cAMP formation	Loss of a putative PKC phosphorylation site	ND	Alexandre et al. (2002)

*Information relating to biochemical properties obtained in specific vertebrate species are marked by the asterisk symbol.

The terminology used in independent studies can be somewhat perplexing as different names were designated to PAC1 isoforms corresponding to the same splicing events identified in various species. In the following section we describe the various splice variants and indicate when a different (i.e., specie-specific) name was designated to the same isoform. A splice isoform that includes all N-terminal encoding exons but does not contain IC (IC3) insertion(s) is referred to as “PAC1-null” (Table 1). The ligand binding and signaling properties of PAC1-null are often used as a reference when assessing the activities of other splice variants.

### N-terminal variations

The N-terminal variants were identified in rodents and humans but not in fishes to date. They are generated by alternative splicing of exon 5 (PAC1-δ5), exons 5–6 (PAC1-short or PAC1-δ5,6), or exons 4–6 (PAC1-very short or PAC1-δ4,5,6) leading to deletions of 7, 21, or 57 amino acids, respectively (Journot et al., 1994; Pantaloni et al., 1996; Chatterjee et al., 1997; Dautzenberg et al., 1999; Lutz et al., 2006; Ushiyama et al., 2007, 2010). In the rat, an insertion of 24 amino acids is caused by splicing of a novel exon 3a located between exons 3 and 4 (PAC1-3a) (Figure 3) (Daniel et al., 2001; Ajpru et al., 2002; Pilzer and Gozes, 2006a). N-terminal splicing isoforms of PAC1 display alterations both in ligand-binding selectivity and coupling to second messengers compared to PAC1-null.

### Intracellular loop variations

PAC1 splice variants in the third IC loop (IC3) have been identified in human, amphibian, fishes, and rodents. These splice isoforms are characterized by the presence of one or two cassettes of 84 nucleotides (hip or hop1 variants) or 81 nucleotides (hop2 variant), or a combination of alternative spliced “cassettes” (hip-hop1 or hip-hop2) (Spengler et al., 1993; Journot et al., 1995; Grimaldi and Cavallaro, 1999; McCulloch et al., 2001; Ronaldson et al., 2002; Fradinger et al., 2005; Ushiyama et al., 2007; May et al., 2010; Holighaus et al., 2011). In human, hip and hop variants are also referred to as SV1 and SV2 or SV3 for hip-hop1 (Pisegna and Wank, 1996; Pisegna et al., 1996). In addition, a splice variant formed by a C-terminal deletion of 193 nucleotides (denoted “*hop1 novel*”) including two amino acids that are essential for G-protein recognition was identified in rat cochlea samples (Abu-Hamdan et al., 2006). Similar alternative splicing variants of PAC1 are also found in non-mammalian vertebrates such as the frog and bony fishes. Three alternative splice variants were identified in the frog IC3 loop (Alexandre et al., 2002). PAC1-R25, with an insertion of 25 amino acids, corresponds to the mammalian hop cassette. PAC1-R41 contains a cassette with no homology to any other variant. PAC1-RMc with a unique cytoplasmic insertion of 13 amino acid into the TM7 domain is missing a canonical Gs recognition motif. PAC1 genes were also identified in zebrafish, goldfish, stickleback, fugu, sea bream, and several others bony fish species. Three zebrafish PAC1 alternative splice variants with insertions in the IC3 loop were identified. Two of them were found to be homologous to the hop1 (84 nucleotides) and hop2 (81 nucleotides) mammalian isoforms (Fradinger et al., 2005). A unique 107 nucleotides inclusion with a premature stop codon (PAC1-skip) resulting in a truncated PAC1 protein did not correspond to any previously detected PAC1 isoform (Fradinger et al., 2005). This isoform resembles an alternative splicing event in a human gene encoding another secretin family receptor, *VPAC1*. Interestingly, the hip cassette variant was not identified in the mouse and zebrafish genomes. Moreover, teleosts genomes contain two (duplicated) PAC1 paralogs, *pac1a* and *pac1b*, however, only *pac1a* encodes for the hop splicing cassette.

### Transmembranal domain variations

A PAC1 variant, cloned from the rat cerebellum, has amino acids deletion/substitution in the TM4 domain along with two amino acid substitutions in the N-terminal (D136N) and TM2 (N190D) domains (Chatterjee et al., 1996; Ajpru et al., 2002). The exact molecular mechanism underlying these variations remains unclear. Notably, the TM4 domain of the secretin family receptors are involved in homo- and hetero-oligomerization of these receptors, associations with receptor activity-modifying proteins (RAMPs), and with GPCR kinases (GRKs) thereby suggesting that splice alterations in the PAC1-TM4 domain may affect receptor function (Morfis et al., 2003; Ritter and Hall, 2009; Magalhaes et al., 2012).

### 5′ UTR variations

Alternative splicing events in exons located at the 5′ UTR were identified for rat *PAC1* gene (Chatterjee et al., 1997). These include different alternative usage of exons located upstream to the ATG translation start codon. Such variations in the 5′ UTR organization and sequences may play a role in the regulation of mRNA expression.

## Alternative PAC1 Splicing Alters Ligand-Binding Properties

PAC1 is considered as being the high-affinity receptor for PACAP, while it displays low binding affinity to the vasoactive intestinal polypeptide (VIP) (Apostolakis et al., 2005; Vaudry et al., 2009; Harmar et al., 2012). Alternative splicing of PAC1 results in different protein products displaying different ligand-binding properties that may result in changes in affinity and selectivity (Table 1). Most studies use the PAC1-null (McCulloch et al., 2001; Holighaus et al., 2011) isoform as a reference for PACAP and VIP binding properties.

Modeling of ligand-receptor binding proposes that the C-terminal part of the ligand PACAP binds to the N-terminus of the PAC1 receptor and that the N-terminal part of PACAP binds to the receptor’s EC loops and TM domains (Furness et al., 2012). Consequently, alterations in the EC domain of PAC1 are predicted to affect these ligand-receptor binding properties (Table 1). Thus, PAC1-very short (a.k.a. PAC1-δ4,5,6), which lacks 57 amino acids in the EC1 domain displays decreased affinity to PACAP27 and PACAP38 but its affinity toward VIP remains the same (Journot et al., 1995; Pantaloni et al., 1996; Dautzenberg et al., 1999; Lutz et al., 2006). The binding affinities for PACAP38 of the δ5, δ5,6 (a.k.a. short) splice isoforms appeared to be very similar to that of PAC1-null, while δ5,6 isoform has increased affinity toward VIP. The rat-specific PAC1-3a isoform containing a 24 residue N-terminal insertion displays increased affinity to PACAP38 but not to PACAP27 (Daniel et al., 2001; Pilzer and Gozes, 2006a). It should be noted that in all of these examples, PACAP is still the better ligand for PAC1 when compared with VIP (Table 1).

The hop1 and hip-hop1 splice isoforms retain similar binding properties to those of PAC1-null. In contrast, insertions into the IC3 loop, namely hip- and hop2-cassettes lead to the elevation of VIP (Spengler et al., 1993; Pisegna et al., 1996; Pilzer and Gozes, 2006b) and the diminution of PACAP binding affinities, thereby suggesting that the alteration in IC3 causes an inside-out conformational change.

Notably, binding analysis of alternative spliced isoforms, which result from combined changes in the EC and IC protein domains yields interesting results. Thus, a splice variant composed of PAC1-short with the inclusion of hop1-cassette, displays similar PACAP and VIP binding properties to that of PAC1-null and different VIP binding properties than PAC1-short alone (Lutz et al., 2006; Ushiyama et al., 2007, 2010). Another combined splice variant composed of PAC1-δ5 or PAC1-δ5,6 together with the hip cassette had different ligand-binding properties to that of PAC1, PAC1-δ5, or PAC1-hip alone (Lutz et al., 2006) (Table 1). This imply that the IC3 domain contributes to the association of PAC1 with its cognate ligands.

These results may be explained by a model for agonist binding to the corticotropin-releasing hormone (CRH) receptor, which belongs to the same GPCR sub-family of PAC1 (Dong et al., 2005). According to this model the TM6 protein domain plays a structural role in ligand binding. Thus, insertions into IC3 loop, which is located between TM5 and TM6 region may cause a conformational change in TM6 and possibility affect binding of PACAP and VIP to the EC part of the receptor. A proof for this model awaits a solved three-dimensional protein structural of the different PAC1 isoforms.

## Alternative PAC1 Splicing Alters Intracellular Signal Transduction

**Figure 4:**
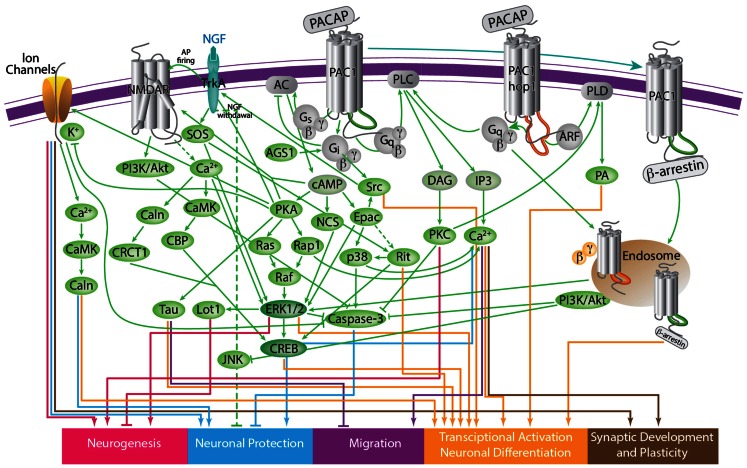
**A scheme depicting the current knowledge on PAC1-mediated signaling cascades resulting in a variety of neuronal outcomes**. PKA was shown to induce ERK1/2 thereby contributing to PACAP neuroprotective (Villalba et al., 1997; Vaudry et al., 2003; Falluel-Morel et al., 2006; Stumm et al., 2007) and neurotrophic (Ravni et al., 2006; Botia et al., 2007; Monaghan et al., 2008) functions. Action potential (AP) firing induces a CRCT1/CREB mediated neuroprotective effect, presumably through NMDA receptor activation and was shown to be initiated by PKA activation (Baxter et al., 2011). NMDA receptor was also shown to be indirectly activated by PACAP (Llansola et al., 2004) or cAMP/PKA signaling (Costa et al., 2009). PAC1/PKA signaling controls cellular apoptosis through inhibition of potassium channels (Mei et al., 2004; Castel et al., 2006; Pugh and Margiotta, 2006) or induction of calcium channels (Pugh and Margiotta, 2006). PAC1/PKA-mediated activation of ion channels leads to activity-dependent neuronal differentiation and synaptic plasticity (Lee et al., 1999; Maturana et al., 2002; Grumolato et al., 2003; Nishimoto et al., 2007). The tumor suppressor gene Lot1 known as a negative regulator of neuronal precursor proliferation was shown to be controlled by PAC1/cAMP/ERK signaling pathway (Fila et al., 2009). PAC1/PKA-dependent phosphorylation of Tau is involved in the control of granule cell migration during cerebellar development (Falluel-Morel et al., 2006). PAC1-mediated cAMP/ERK-dependent neurite outgrowth was shown to be regulated via a novel neuritogenic factor NCS (Emery and Eiden, 2012) or Epac/ERK (Gerdin and Eiden, 2007) pathways. PAC1/Epac was shown to regulate neuronal differentiation via activation of p38 kinase along with mobilization of Ca^2+^ from intracellular stores (Ster et al., 2007) as well as Epac/Rit-dependent pathway involving CREB signaling (Shi et al., 2006). PAC1 was demonstrated to induce Rit through TrkA/Shc/SOS signaling initiated by Src activation via dual Gs/Epac and Gi stimulation (Shi et al., 2010). PACAP also signals through Gq-linked PLC > IP3 > Ca^2+^/DAG > PKC, and PLD > phosphatidic acid (PA) pathways (Journot et al., 1994; Makhlouf and Murthy, 1997; Dejda et al., 2006). PAC1 is a mediator of gene transcription, neuronal differentiation, and synaptic development (Masmoudi-Kouki et al., 2006; Vaudry et al., 2007; Andero and Ressler, 2012). As an example of functional diversity caused by PAC1 alternative splicing two additional PAC1-hop1 signaling pathways are presented on the scheme. They depict PAC1-hop1/ARF dependent PLD activation (McCulloch et al., 2001) and internalization-dependent engagement of PI3Kγ/Akt activation (May et al., 2010). AC, adenylate cyclase; AGS, activator of G protein signaling; Akt, serine-threonine protein kinase PKB; cAMP, cyclic adenosine monophosphate; ARF, ADP(adenosine diphosphate) ribosylation factor; Caln, calcineurin; CaMK, calcium calmodulin kinase; CBP, creb binding protein; CRCT1, cysteine-rich C-terminal 1; CREB, cAMP responsive element-binding protein; DAG, diacyl glycerol; Epac, exchange factor directly activated by cAMP; ERK, extracellular signal-regulate kinase; G, guanine nucleotide-binding regulatory protein; IP3, inositol-1,4,5-triphosphate; JNK, c-Jun oncogene N-terminal kinase 1; Lot1, lost on transformation 1; NCS, neuritogenic cAMP sensor; NGF, nerve growth factor; NMDAR, *N*-methyl-d-aspartate receptor; p38, p38 mitogen-activated protein kinase; PA, phosphatidic acid; PI3K, phosphatidylinositol 3′ OH kinase; PKA, protein kinase A; PKC, protein kinase C;PLC, phospholipase; Raf, B-Raf proto-oncogene serine/threonine kinase;Ras, retrovirus-associated DNA sequences; Rap1, Rit, small GTPases of the RAS oncogene family; Sos, son of sevenless homolog 1; Src, sarcoma viral oncogen homolog; Tau, neuron-specific microtubule-associated protein; TrkA, tropomyosin-related kinase.

Ligand binding to PAC1 results in allosteric changes in the IC docking sites for effector coupling. A variety of IC signal transduction cascades can be differentially transduced by multiple PAC1 splice variants with altered EC or IC protein domains (Figure 4) (Arimura, 1998; Dickson and Finlayson, 2009; Vaudry et al., 2009; Furness et al., 2012). The most studied pathways include coupling of PAC1 to Gs and Gq proteins and activation of AC and phospholipase Cβ PLCβ) which result in the respective stimulation of production of cAMP and/or IP3 (Milligan and Kostenis, 2006; Couvineau and Laburthe, 2012) (Figures 3 and 4). In addition, PAC1 was shown to regulate the level of IC Ca^2+^ in either G-protein dependent or independent manners. The following section reviews the role of PAC1 splice variants in the activation of the above pathways as well as other non-canonical signaling cascades (see also Table 1).

### AC and PLCβ

When compared to the PAC1-null isoform, some alternative spliced variants display altered mode of signaling. For example, PAC1 isoform 3a, δ4,5,6 (a.k.a. very short), hip, hip-hop, δ5-hip, and δ5,6-hip show reduction in the potency of agonists-mediated cAMP and IP3 production (Spengler et al., 1993; Dautzenberg et al., 1999; Parsons et al., 2000; Daniel et al., 2001; Germano et al., 2004; Lutz et al., 2006; Pilzer and Gozes, 2006a; Holighaus et al., 2011). Similarly, PAC-TM4, hop1 novel, δ5,6,14–17, skip, and Rmc have lost their ability to stimulate cAMP and IP3 production (Chatterjee et al., 1996; Daniel et al., 2001; Ajpru et al., 2002; Alexandre et al., 2002; Fradinger et al., 2005; Abu-Hamdan et al., 2006; Pilzer and Gozes, 2006a). Notably, all of the above splice variants have modified or deleted IC3, TM, and C-terminal protein domains that are known to be important for G-protein binding (Chatterjee et al., 1996).

In some studies, PAC1-null displays very low or no activation of PLCβ while PAC1-hop1 switches its mode of signaling from AC to PLCβ (Spengler et al., 1993; DiCicco-Bloom et al., 2000; Nicot and DiCicco-Bloom, 2001; Ronaldson et al., 2002; May et al., 2010). For example, PAC1-hop1 was reported to mediate the activation of both AC and phospholipase C signaling in cortical sympathetic neuroblasts, while PAC1-null merely signals via AC stimulated pathway (Braas and May, 1996; DiCicco-Bloom et al., 2000). Other studies report that both isoforms display dual activation of AC and PLCβ pathways, however the hop1 isoform has higher activation of these pathways. Thus, analyses of SV1/hip and SV2/hop1 show that these isoforms display increased coupling to cAMP and PLCβ, respectively (Spengler et al., 1993; Pisegna and Wank, 1996; Lu et al., 1998; Braas and May, 1999; Parsons et al., 2000; Nicot and DiCicco-Bloom, 2001; Germano et al., 2004; Lutz et al., 2006; May et al., 2010; Holighaus et al., 2011). The frog R25 and R41 isoforms containing amino acid insertions into IC3 have higher activation of cAMP compared to PAC1-null (Alexandre et al., 2002).

Consistent with the effect of IC3 loop insertions on ligand-binding properties, hip- and hop2-cassettes conferred comparable potency to induction of cAMP and IP3 accumulation by PACAP and VIP but at the same time characterized by much lower efficacy of this response (Spengler et al., 1993; Pisegna and Wank, 1996; Pisegna et al., 1996; Ronaldson et al., 2002; Lutz et al., 2006). On the other hand, PAC1-hip variant was shown to retain AC mediated signaling but demonstrated impaired coupling to the PLCβ pathway (May et al., 2010).

### Ca^2+^signaling

The concentration of calcium ions (Ca^2+^) in the cytoplasm is controlled by its uptake and release by specific transporter proteins residing in the plasma membrane, the inner mitochondria membrane, and the endoplasmic reticulum (ER) (Clapham, 2007). PAC1-mediated Ca^2+^ signaling was shown to play important roles in regulating neurotransmitter release, and neurotransmitter receptors (Shioda et al., 1997; Taupenot et al., 1999; Germano et al., 2009; Mustafa et al., 2010; Pugh et al., 2010; Amir-Zilberstein et al., 2012; Smith and Eiden, 2012). The effects of PAC1 splice isoforms on the levels of cytoplasmic Ca^2+^ were reported in the cases of PAC1-null hop1/2, δ5,6/short, 3a, and PAC1-TM4 (Table 1) (Dautzenberg et al., 1999; Lutz et al., 2006; Mustafa et al., 2007, 2010; Nishimoto et al., 2007; Germano et al., 2009; Hansson et al., 2009; Vallejo, 2009; Ushiyama et al., 2010; Holighaus et al., 2011). PACAP is known to modulate both EC Ca^2+^ influx via voltage-gated calcium channels (VGCC) and Ca^2+^ release from IC ER stores through both IP3/PLCβ and AC pathways but also through other signaling cascades (Tanaka et al., 1997; Shioda et al., 1998; Grimaldi and Cavallaro, 1999).

PAC1-null was reported to modulate cytosolic Ca^2+^ mobilization from both EC and IC stores (Shioda et al., 1997; Masmoudi et al., 2003; Payet et al., 2003; Nishimoto et al., 2007). In acutely dissociated rat melanotrophs, the increase of cytosolic Ca^2+^ was dependent on the activation of non-selective cation channels and the facilitation of voltage-dependent Ca^2+^ channels by PKC- and PKA-dependent phosphorylation, respectively (Tanaka et al., 1997). cAMP-dependent entry of EC calcium was also reported in astrocyte cells (Vallejo, 2009). It was also suggested that PAC1-null-mediated elevation of cytoplasmic Ca^2+^ levels in NG108-15 cells is due to IP_3_ receptor-mediated Ca^2+^ release from IC stores (Holighaus et al., 2011).

The PAC1-hop1 variant is responsible for both Ca^2+^ mobilization from IC stores and influx through voltage-gated Ca^2+^ channels in bovine chromaffin cells (Mustafa et al., 2007). Transfection of PAC1-hop to the adrenomedullary pheochromocytoma (PC12) cell line showed sustained IP_3_-mediated Ca^2+^ release from IC stores and from store-operated Ca^2+^ entry (SOCE) (Taupenot et al., 1999; Mustafa et al., 2007, 2010). In bovine adrenal chromaffin cells, PAC1-hop was shown to mediate cytosolic Ca^2+^ release from ryanodine/caffeine-sensitive Ca^2+^ stores that was not dependent on either cAMP or IP3 generation (Tanaka et al., 1998; Payet et al., 2003). Heterologous expression of the rat PAC1-hop1 but not PAC1-hip variant in NG108-15 and PC12 cells leads to an increase in IC Ca^2+^ concentration (Mustafa et al., 2007; Holighaus et al., 2011). In both cell lines the response consisted of a rapid and transient rise of Ca^2+^ reminiscent of IP_3_ receptor-mediated Ca^2+^ release from IC stores followed by a prolonged Ca^2+^ accumulated from EC source.

When compared with PAC1-null, PAC1-hop1 was more potent in Ca^2+^ elevation (Holighaus et al., 2011). However, following overexpression in Chinese Hamster Ovarian cells, activation of PAC1-null induced higher Ca^2+^ levels when compared with activation of PAC1-hop1 (Ushiyama et al., 2010). PAC1-hop1 also mediated PACAP-induced Ca^2+^ release from ER and PKCγ translocation to the nucleus and plasma membrane resulting in the astrocytic differentiation (Nicot and DiCicco-Bloom, 2001). Finally, stimulation of voltage-gated L-type or non L-type Ca^2+^ channels following initiation of PAC1-hop1 signaling was also reported (Mustafa et al., 2010).

Somewhat limited data was reported with regards to Ca^2+^ signaling via other PAC1 splice variants. Thus, δ5,6/short as well as the PAC1 isoform containing combined “short” deletion in the N-terminal EC1 domain along with hop1-cassette in the IC3 loop exhibit PACAP-induced cytoplasmic calcium elevation (Ushiyama et al., 2010). PAC1-3a was shown to induce Ca^2+^ accumulation through coupling to Gs/cAMP rather than Gq/PLCβ pathway (Pilzer and Gozes, 2006a). PAC1-TM4 that was found to be inactive when assayed for both cAMP and PLCβ activation but it displayed elevation of Ca^2+^ in response to PACAP27. It was demonstrated that this effect involved the modulation of voltage-gated L-type Ca^2+^ channels (Chatterjee et al., 1996).

Taken together, the apparent inconsistent results concerning the mechanisms underlying the aforementioned Ca^2+^ signaling events are most likely due to the different cellular systems (e.g., cell types) employed by the above studies to analyze PAC1-mediated IC Ca^2+^ changes.

### Other transduction pathways

Signaling of PAC1 through interaction with cytoplasmic protein partners, other than the canonical Gs and Gq, was reported mainly in the case of the PAC1-hop1 isoform (McCulloch et al., 2001; Ronaldson et al., 2002). Both PAC1-null and -hop1 proteins were reported to activate phospholipase D (PLD). Although PAC1-null-mediated PLD stimulation involved Gq/11 > PLC > PLD pathway, PAC1-hop1 was capable of activating PLD through direct binding to ADP-ribosylation factor (ARF) (McCulloch et al., 2002; Dejda et al., 2006).

## Expression of PAC1 Splice Isoforms in the Nervous System

Information on time- and region-specific distribution of PAC1 splice isoforms may shed light on how PAC1 gene products regulate a plethora of biological functions in developmental and physiological processes (D’Agata et al., 1996; Waschek et al., 2000; Waschek, 2003; Vaudry et al., 2009). The distribution of PAC1 has been examined in many species using different techniques that revealed widespread expression in different tissues, including the nervous, cardiovascular, endocrine, immune, and respiratory systems (Aino et al., 1995; Abu-Hamdan et al., 2006; Gomariz et al., 2006; Molnar et al., 2008; Jolivel et al., 2009; Shneider et al., 2010; Lugo et al., 2011; Buljan et al., 2012). However, knowledge concerning the expression of PAC1 splice variants is somewhat limited at it is mainly based on analyses of isolated brain areas and/or primary cell cultures. The reported changes in PAC1 receptor variant expression appear to be most evident during development. We have summarized the existing data concerning the expression of these variants by focusing on expression in the nervous system of different mammalian species.

The major mRNA isoform of PAC1 in the brain is PAC1-null, which contains no splice deletions or insertions. PAC1-null is predominantly expressed in neurons residing in different brain areas although it is also detected in glial cells of the cerebella cortex and in activated astrocytes (Pilzer and Gozes, 2006b; Dickson and Finlayson, 2009; Vaudry et al., 2009).

PAC1 variants with N-terminal deletions are well represented in human fetal brain tissues and in five human neuroblastoma lines (Lutz et al., 2006; Falktoft et al., 2009), thereby suggesting their role in immature nervous tissue. N-terminal splice variants were detected in different brain areas. The PAC1-short variant with 21 amino acids N-terminal deletion was predominantly found in the thalamus, hypothalamus, and the hypophysis, and more moderately in the amygdala and retina (Dautzenberg et al., 1999; Grimaldi and Cavallaro, 1999; Jamen et al., 2002; Girard et al., 2004; Lutz et al., 2006; Ushiyama et al., 2007, 2010; Hammack et al., 2010a). It was shown to be expressed in cochlea subfractions along with the PAC1-δ5 isoform with a seven amino acid deletion (Abu-Hamdan et al., 2006). The PAC1-very short variant (57 amino acid deletion) was also detected at low levels in the mouse amygdala and cortex though not in the retina. All three N-terminal variants (null, short, and very short) were abundantly expressed in neuronal tissues (Dickson and Finlayson, 2009; Vaudry et al., 2009). The PAC1-3a variant containing N-terminal insertion of 24 amino acids was detected in rat cerebral cortex. This splice isoform is also highly expressed in distinct cell populations of the testis, including Sertoli cells, pachytene spermatocytes, and round spermatids (Daniel et al., 2001; Ajpru et al., 2002; Pilzer and Gozes, 2006a).

Contrary to the aforementioned N-terminal splice variants that were only reported in mammals, IC loops (i.e., IC3) splice products are found in all studied vertebrate species. In the postnatal cerebral cortex of rats, the expression of PAC1-null, hop1, hip, and hip-hop1 dramatically decreases during the first neonatal month suggesting a major role for these isoforms in embryonic development (Shneider et al., 2010). Interestingly, the expression of these isoforms was shown to be gender dependent displaying higher levels in female postnatal brain at least during the first 3 months of development. In the developing retina, the proportion of IC3 PAC1 isoforms changes as development proceeds: PAC1-hip-hop1 transcript demonstrated transient elevation at day P10, while a decrease in PAC1-null and hip along with elevation of PAC1-hop1 level was observed by P20 (Lakk et al., 2012). PAC1-hop1 expression was found in the olfactory bulb, hippocampus, cerebral cortex, cerebellum, and striatum of newborn rats. PAC1-hop2 was present in rat cerebral astrocytes from newborn brains, in neuronal enriched cultures, and in PC12 cells that undergo neuronal differentiation following NGF treatment (Jamen et al., 2002; Onoue et al., 2002; Hashimoto et al., 2003; Ravni et al., 2006; Mustafa et al., 2010).

PAC1-hip was detected at much lower levels in adult tissues and it was therefore speculated to be important for the embryonic development (Shneider et al., 2010; Holighaus et al., 2011). The relative expression of human and rat PAC1 splice variants in the frontal cortex was reported to have the following expression level hierarchy: null > SV1/hip > SV2/hop = SV3/hip-hop (Pisegna and Wank, 1996; Pisegna et al., 1996; Germano et al., 2004; Lutz et al., 2006). PAC1-hop1 was found to be expressed in all neuroendocrine cells, suggesting a fine tuning of PAC1-mediated signaling in the neuroendocrine cells (Mustafa et al., 2010). PAC1-hop1 splice isoform was the major form expressed in the superior cervical ganglia (SCG) sympathetic neurons, which also express PAC1-short (Braas and May, 1999). Zebrafish IC3 splice isoforms are widely expressed in the adult tissues with PAC1-hop1 detected in brain and testis, PAC1-hop2 in the ovary and PAC1-skip variant in the gills (Fradinger et al., 2005). Lastly, although PAC1-TM4 isoform is mainly found in pancreatic β-cells it is also expressed in the cerebral cortex, cerebellum, and brain stem (Chatterjee et al., 1996; Ajpru et al., 2002).

In summary, changes in PAC1 receptor variant expression appear to be most evident during development. The fact that PAC1 splice variants display differential expression patterns in the nervous system suggests that alternative splicing of this gene product plays a role in fine tuning of PAC1 activity in these areas.

## Regulation of PAC1 Splicing by Neuronal-Specific RNA-Binding Proteins

**Figure 5:**
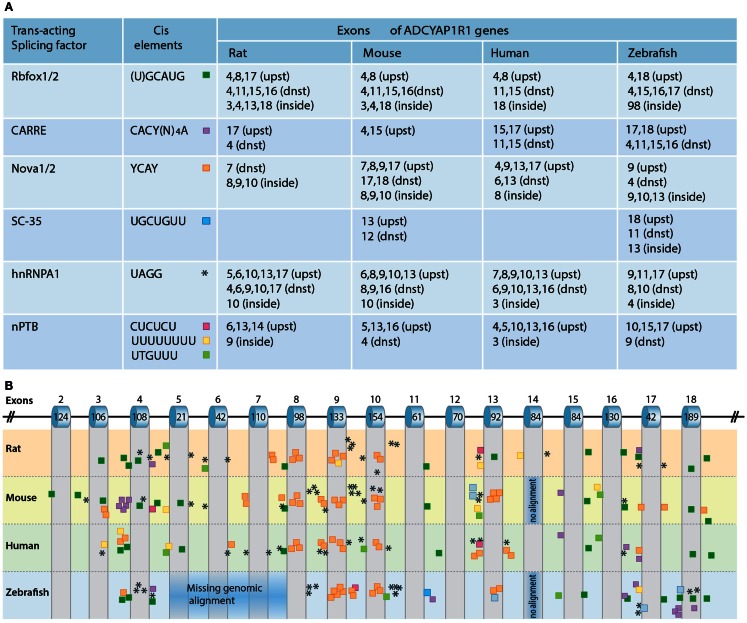
**Regulation of PAC1 splicing**. **(A)** A chart depicting the predicted binding sites for neuronal-specific RNA-binding proteins that potentially regulate PAC1 gene splicing by binding in proximity to PAC1 exons. Consensus cis-acting binding DNA elements for the respective RNA-binding protein are depicted with respect to their position in the PAC1 genes of rat, mouse, human, and zebrafish PAC1. We analyzed the presence of putative consensus binding sites located within 300 base-pairs upstream (upst) or downstream (dnst) and inside the exons. **(B)** Schematic representation of the PAC1 gene in which the location of the analyzed cis-acting elements is color-coded as above. Corresponding exons numbers and sizes (bp) are indicated.

The regulation of PAC1 splicing is not well understood. Alternative splicing of pre-mRNA is often regulated by conserved cis-regulatory RNA sequence elements that serve as recognition sites for different splicing factors. These RNA-binding splicing factors differ from the general spliceosome machinery proteins due to the formers’ ability to either activate or repress the inclusion of alternatively spliced exons, depending on whether the factors’ binding site is located upstream, downstream, or inside the exon. Neuronal-specific splicing factors include nova-1/2, Rbfox-1/2, and ELKV2 splicing factors, the neuronal-specific polypyrimidine tract-binding protein (nPTB) as well as a set of heterogeneous nuclear ribonucleoproteins (hnRNP A1,L,F.H1). As the consensus cis-acting binding sequences for the aforementioned splicing factors have been identified, their involvement in regulating the alternative splicing of a given gene by sequence analysis can be predicted. We analyzed the presence of known consensus binding sites for the following RNA-binding proteins: Rbfox-1/2, Nova-1/2 (single and repeatedly organized elements), SC-35, nPTB enhancers and silencers, and CaRRE for Ca^2+^/CaM kinase IV recognition sequences. Putative binding sites for neuronal-specific splicing factors in the respective PAC1 genes of human, mouse, and zebrafish are shown in Figure 5.

Rbfox-1/2 can act either as a splicing enhancer or as an inducer of exon skipping (Zhang et al., 2008). Putative Rbfox-1/2 binding sites located within a short distance downstream of target exons are predicted to induce exon inclusion whereas upstream binding sites are predicted to cause exon skipping. Such Rbfox-1/2 recognition element located within a short distance downstream of hop1 encoding exon has been experimentally validated as a genuine Rbfox-1 binding site (Lee et al., 2009). Our own PAC1 bioinformatic analysis identified a putative Rbfox-1/2 binding element within −30 nucleotides downstream from conserved exon spanning zebrafish TM7 suggesting that this domain might be regulated by alternative splicing (Figure 5). Rbfox-1/2 motifs are also present in the sequences of all examined species downstream of exon 16 encoding parts of the TM7 domain. Another recognition site is located upstream of exon 4 consistent with a deletion of the exon resulting in the generation of PAC1-very short splice variant (Figure 5). Rbfox-1/2 recognition motif predicts potential deletion of exon 8 that may result in a generation of a soluble PAC1 receptor as in the case of the CRH receptor (Zmijewski and Slominski, 2009, 2010). Rbfox-1/2 binding site is also detected downstream of exon 11, which is known to encode the most hypervariable amino acid sequence among Secretin family GPCRs, as well as part of TM4 domain which undergoes alternative splicing in PAC1-TM4 isoform (Markovic and Grammatopoulos, 2009).

Calcium signaling plays an important role in neuronal development and is involved in the regulation of immediate and long-term neuronal responses to various stimuli such as stressors and hormones (Ghosh et al., 1994; West et al., 2001). Depolarization is known to be critical for modulating the neuronal activity that induces Ca^2+^-dependent gene regulation, including alternative splicing. Ca^2+^-dependent splicing is mediated by L-type calcium channels and by Ca^2+^/calmodulin-dependent protein kinases IV (CaMK IV) (Lee et al., 2007, 2009). The latter was shown to repress splicing of target genes containing specific recognition elements (CaRRE) located within the 3′ splice site or inside the exon. Our own analysis detected additional putative CaRREs that can be responsive to CaMK IV. We predicted single, multiple, and tandemly organized CaRRE repeats upstream of exon 15 and 17 of the zebrafish, human, and mouse PAC1 receptors, suggesting the involvement of CaMK IV in regulating the alternative splicing of these exons. Putative CaRREs are also present proximal to mouse, rat, and zebrafish exon 4 (Figure 5).

Exonic and intronic hnRNPA1 and nPTB cis-elements that are important for activity-dependent splicing in neuronal cells were shown to repress the inclusion of target exons (Allemand et al., 2005; An and Grabowski, 2007; Donev et al., 2007; Resnick et al., 2008; Zheng et al., 2012). Putative hnRNPA1 and nPTB recognition sites are located proximal to exons 4, 5, and 6 consistent with the known deletions of these exons that result in the generation of PAC1-short and very short splice variants. Putative hnRNPA1 recognition motifs are also found near exons 8–10 encoding to IC1, IC2, and EC2 domains (Figure 5).

Nova-1/2 regulates target exon inclusion or skipping with broad distribution of binding sites across the gene sequence (Ule et al., 2005; Yano et al., 2010; Norris and Calarco, 2012). Predicted Nova-1/2 recognition elements are found in human and mouse PAC1 and in both paralogs of zebrafish PAC1 gene (1a and 1b). Interestingly, higher frequency of putative splicing-inducing AU-rich and Nova-specific sequences are found in intronic regions upstream of alternative spliced exons characteristic of the Secretin family splicing patterns, e.g., around exon 4, 8, 9, 10 (Zmijewski and Slominski, 2009) as well as exon 17. Exons 4–6 encode sequences covering known N-terminal PAC1 deletion regions (deletions of 7, 21, and 57 amino acids). Exon 17 encodes the last amino acids of the seventh TM domain and the beginning of the IC tail shown to represent the highly conserved exon within the secretin family (Markovic and Grammatopoulos, 2009). Skipping of this exon was not detected in the PAC1 receptor gene, but it has been proposed that such exon skipping in the human CRF1 and PTH1 receptors results in a six TM receptor displaying impaired trafficking (Shyu et al., 1996; Grammatopoulos et al., 1999; Markovic et al., 2008). The rabbit calcitonin receptor is also formed as a result of the skipping of the TM7 domain and displays slightly diminished cAMP activation along with deficiency in IP3 induction (Shyu et al., 1996). Taken together the above analysis of potential cis-elements spanning TM7 exons predicts that a PAC1 splice variant containing TM7 skipping may exist in neuronal tissues.

An important question raised in this chapter is whether the aforementioned RNA-binding factors regulate PAC1 splicing *in vivo*. It has been demonstrated that alternative splicing of the PAC1 receptor is regulated by the Rbfox-1 splicing factor in depolarized neurons. In this case, neuronal depolarization induced CaM-kinase dependent self-splicing of Rbfox-1, leading to the translocation of Rbfox-1/2 from the cytoplasm to the nucleus where Rbfox-1 could mediate the alternative splicing of neuronal-specific target genes, including PAC1. Interestingly, Rbfox-1 mRNA levels were shown to be regulated by the Orthopedia (Otp) homedomain-containing protein. The latter is a hypothalamic-specific transcriptional factor, which plays a role in hypothalamic neuronal-specification during development and in regulation of body homeostasis in the mature zebrafish brain (Acampora et al., 1999; Wang and Lufkin, 2000; Blechman et al., 2007; Ryu et al., 2007; Amir-Zilberstein et al., 2012; Fernandes et al., 2012). The involvement and regulation of other neuronal-specific RNA-binding factors in PAC1 splicing remains to be determined.

## Pleiotropic Roles of PAC1 Splice Isoforms in the Nervous System

The distribution of PAC1 and its ligands PACAP and VIP in a variety of cell types is reminiscent of the pleiotropic functions of PAC1 in development and physiology. PACAP is described in the literature as a hormone, neuropeptide, endocrine peptide, neurotransmitter, and neurotrophic factor. It affects the central nervous system (CNS), cardiovascular system, pituitary, thyroid, and adrenal glands and provides functional activities in the gonads, gastrointestinal tract, and pancreas (Waschek et al., 2000; Dickson and Finlayson, 2009; Vaudry et al., 2009). In the nervous system, PAC1 was shown to affect a variety of hormones and neuropeptides, including stimulation of oxytocin (Jamen et al., 2003) and melatonin (Nakahara et al., 2002) release and *de novo* mRNA synthesis of CRH (Nakahara et al., 2002; Amir-Zilberstein et al., 2012), arginine-vasopressin (Murase et al., 1995; Gillard et al., 2006), GnRH (Kanasaki et al., 2009; Winters and Moore, 2011), somatostatin (Kageyama et al., 2007), and MSH (Mounien et al., 2006). This overabundance of PAC1-mediated physiological activities can be made possible through the signaling diversity of its alternatively spliced gene products. Moreover, different expression of PAC1 isoforms is common in neuronal ontogeny. Such differences in the expression of PAC1 splice variants might modulate final outputs of VIP and PACAP activities as neurotransmitters, neurotrophic, or differentiation factors. Examples for the involvement of PAC1 splice isoforms in mediating these activities are described below.

### Neurogenesis, neuroprotection, and differentiation

Neural progenitor cells (NPC) and astroglial cells express PAC1-null and PAC1-hop1 variants that mediate both cAMP- and Ca^2+^-dependent signaling pathways and induce production of a subset of neurotrophic factors resulting in neuronal proliferation and/or differentiation. PAC1-hop2 variant, detected in astrogenic and neuronal populations, promote neuroprotective function induced by VIP. In another study, VIP was also demonstrated to induce PAC1-hop2-mediated astrocytes neuroprotection against oxidative stress. Astrocytic expression of PAC1-hop2 isoform may therefore play a critical role in the NPC shift toward neuronal or astrocytes differentiation (Ashur-Fabian et al., 1997; Pilzer and Gozes, 2006b).

Expression of PAC1-null and PAC1-hop1 define region-specific neurogenesis in the CNS and peripheral nervous systems (Lu et al., 1998). Both variants are differently expressed in proliferating sympathetic (PAC1-hop1) and cortical precursors (PAC1-null) revealing opposing PACAP-mediated mitogenic regulation – either by stimulating sympathetic neuroblast proliferation or by inhibiting cortical precursor mitosis. Ectopic expression of PAC1-hop1 in cortical neuroblasts may transform the anti-mitotic effect of PACAP into promitotic. This promitotic signaling was shown to involve PLC signaling pathway (Nicot and DiCicco-Bloom, 2001). PAC1-hop1 was also shown to promote sympathetic neurons survival following growth factor withdrawal (May et al., 2010).

In conjunction with the abovementioned, PACAP may possess therapeutic potential for neurodegenerative pathologies, such as Parkinson’s and Alzheimer’s disease (AD). PACAP is enriched in rat mesencephalic dopaminergic neurons and protects them from neurotoxin-induced death (Reglodi et al., 2004). PACAP was shown to be downregulated in several mouse models for AD as well as in the human temporal cortex of AD patients (Kojro et al., 2006; Rat et al., 2011; Postina, 2012). Amyloidogenic processing of the amyloid precursor protein (APP) to αβ-peptides is responsible for the development of AD. However, non-amyloidogenic APP processing pathway results in the α-secretase-dependent cleavage within the αβ-peptide region, preventing AD pathology. Continual PACAP and PAC1 activation resulted also in a feed-forward autocrine elevation of both PACAP and PAC1 in mice that may further facilitate non-amyloidogenic APP cleavage. α-secretase activation was shown to be regulated by ERK1 and ERK 2 and PI-3 kinase, suggesting that PAC1-hop1 is the most efficient isoform with regard to the activation of these downstream PLC effectors (Kojro et al., 2006; Rat et al., 2011; Postina, 2012). It remains to be determined whether specific PAC1 splice variants are involved in these neuroprotective activities.

### Neurosecretion and neurotransmission

Pituitary AC-activating polypeptide was shown to elicit catecholamine synthesis and release. Expression of PAC1-hop1 in bovine chromaffin NG108-15 cell line, which lacks endogenous PAC1 receptors, induces the release of norepinephrine via a Ca^2+^ influx-dependent mechanism (Mustafa et al., 2007). Transfected PAC1-hop1 triggers sustained catecholamine secretion by regulating Ca^2+^ levels through both ER and EC Ca^2+^ channels (Mustafa et al., 2010; Smith and Eiden, 2012). Moreover, acute and long-term met-enkephalin secretion and enkephalin biosynthesis were attributed to bovine chromaffin cells upon PAC1-hop1-mediated activation of L-type Ca^2+^ channels. This implies a role of hop1 splice cassette in regulating neuroendocrine secretion. SCG sympathetic neurons were shown to predominantly express the PAC1-hop1 splice isoform and PACAP stimulates neuropeptide Y release in these neurons via a mechanism involving both AC and PLCβ (Braas and May, 1999).

## Role of PAC1 Splice Isoforms in Body Homeostasis

PAC1 is implicated in the regulation of homeostatic processes, including food and liquid consumption (Nomura et al., 1997; Mounien et al., 2006, 2009), sleep (Hannibal and Fahrenkrug, 2004), stress (Pilzer and Gozes, 2006b; Amir-Zilberstein et al., 2012), locomotion (Vaudry et al., 2000), memory and learning activities (Dong et al., 2010; Andero and Ressler, 2012; Holighaus et al., 2012), and circadian functions (Ajpru et al., 2002; Hannibal and Fahrenkrug, 2004). Recent studies have indicated that at least some of these activities are modulated by alternative splicing of PAC1 (Ajpru et al., 2002; Hannibal and Fahrenkrug, 2004; Pilzer and Gozes, 2006b; Holighaus et al., 2011, 2012; Amir-Zilberstein et al., 2012).

Pituitary AC-activating polypeptide/PAC1 signaling was recently implicated in abnormal stress responses underlying post-traumatic stress disorder (PTSD) pathology (Ressler et al., 2011; Andero and Ressler, 2012; Hauger et al., 2012). PACAP38 levels in females were strongly associated with PTSD symptoms. Ressler et al. and May et al. (2010) found that a single nucleotide polymorphism (SNP), rs2267735 in the PAC1 gene is correlated with PTSD in a gender-specific estrogen-dependent manner. This SNP, which resides in the putative estrogen response element within PAC1’s promoter/enhancer is associated with sex-dependent PTSD, fear discrimination, PAC1 mRNA expression, and methylation of PAC1. Using mouse models, these authors showed that PAC1 mRNA was induced by fear conditioning or estrogen hormone replacement in the bed nucleus of stria terminalis (BNST), which is a component of the extended amygdala involved in fear- and anxiety-like responses.

In connection to the above, steroid-induced changes in PAC1 splice isoforms were demonstrated in the case of PAC1-hop2 (Apostolakis et al., 2005). PAC1-hop2 and the N-terminal PAC1-short splice variants were detected in the hypothalamic ventromedial nucleus (VMN) upon estradiol or dual estradiol/progesterone treatment. The ratio of PAC1-hop2 mRNA to other PAC1 variants in VMN depends on steroid application, implying its importance for cumulative PAC1 isoforms expression and hence signaling properties. PACAP-induced sexual/mating behavior in female rats was dependent on progesterone receptor in the VMN. Estradiol stimulates synthesis of progesterone, which in turn facilitates PACAP synthesis and activation of PAC1-short and -hop2 signaling critical for the induction of animal sexual receptivity.

**Figure 6:**
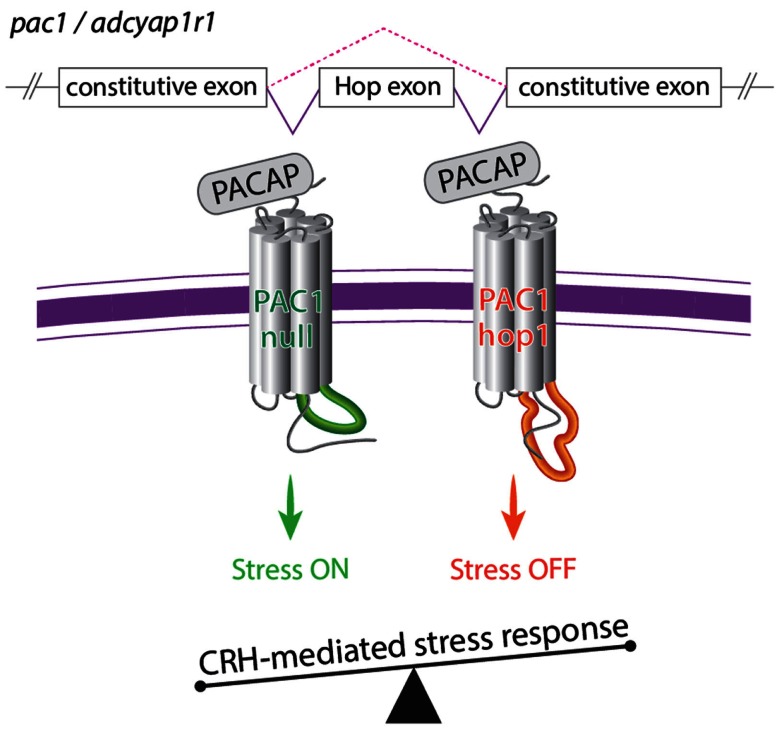
**A model illustrating the role of alternative splicing of PAC1 hop cassette that serves as an ON/OFF stress switch**. In response to various stressors, the so called PAC1-null splice variant (i.e., no deletion in the N-terminal domain and no addition to the intracellular loops) modulate transcriptional activation of CRH and stress behaviors to adapt to the changes in homeostasis. Termination of CRH-mediated stress response is mediated by means of regulation of PAC1 gene splicing and inclusion of an altered exon (hop1) encoding to 28 amino acids of the third intracellular loop leading to the formation of the PAC1-hop1 splice variant. Generation of the PAC1-hop isoform terminates stress response by means yet to be uncovered (see text and Amir-Zilberstein et al., 2012).

PAC1 signaling is known to be required for physiological stress response as activation of PAC1 by PACAP is required for stress-induced CRH transcription *in vivo* and *in vitro* (Agarwal et al., 2005; Kageyama et al., 2007; Hammack et al., 2010b; Stroth and Eiden, 2010; Tsukiyama et al., 2011). The above studies suggest that PAC1 splice variants and their relative expression ratio might be involved in the regulation of body homeostasis including physiological responses to stressor challenges. A recent study performed in our lab, employed various stress paradigms in mouse and zebrafish to demonstrate that activation and termination of CRH transcription caused by stressful stimuli is regulated by an interrelationship of PAC1-null and hop1 isoforms (Amir-Zilberstein et al., 2012). Thus, the generation of PAC1-hop1 by alternative splicing leads to the termination of CRH transcription, normal activation of the hypothalamic-pituitary-adrenal axis, and adaptive anxiety-like behavior (Amir-Zilberstein et al., 2012). We therefore suggested that alternative splicing of the hop1-cassette serves as an ON/OFF stress switch (Figure 6).

PAC1 rs2267735 gene polymorphism is also associated with increased dark-enhanced startle (DES) in adult females but not males with PTSD (Ressler et al., 2011). Moreover, children of abused mothers show elevated DES and the same PAC1 gene polymorphism associated with PTSD risk in adult females is also associated with increased DES in these children (Jovanovic et al., 2011, 2012). Notably, zebrafish larvae show a strong aversion to the dark side of a two-compartment light/dark arena and this place preference can be mitigated by anxiolytic drugs, such as Diazepam, indicating that this assay measures an anxiety-like behavior (Steenbergen et al., 2011; Schnorr et al., 2012). We found that during the recovery phase that follows a stressful challenge (osmotic shock), wild type larvae decrease their dark-avoidance time, a phenotype indicative of decreased stress-related anxiety, while larvae with depleted PAC1a-hop1 display delayed dark-avoidance recovery (Amir-Zilberstein et al., 2012). Thus, the delayed behavioral response of PAC1a-hop1-depleted embryos correlates with their respective failure to terminate *crh* and cortisol levels following stressors.

## Conclusion

Alternative splicing is a major gene regulatory process involving cis- and trans-acting factors. PAC1 signaling controls a variety of cellular and physiological responses, such as differentiation, proliferation, cell cycle regulation, neurotransmitter, and hormone release and adaptation to stressful challenges. The PAC1 gene encompasses a relatively long genomic region, which consists of up to 18 exons and contains many putative splicing factors recognition sites that might be activated during different phases of neuronal activation. PAC1 receptor signaling can be fine-tuned by the generation of a set of alternatively spliced variants produced in a spatio-temporal manner. Splicing-dependent alterations in PAC1 protein domains modify its ligand binding and signaling properties leading to a range of cellular activities. Although the physiological function(s) of the vast majority of the alternatively spliced PAC1 gene products is still unknown, recent studies have implicated certain PAC1 splice variants in the regulation of homeostatic processes such as adaptation to stressful challenges. In view of the recent association of PAC1 with PTSD risk, the regulation of PAC1 splicing and its underlying physiological outcomes might prove to be relevant to the etiology of some neurological and psychiatric disorders.

## Conflict of Interest Statement

The authors declare that the research was conducted in the absence of any commercial or financial relationships that could be construed as a potential conflict of interest.
